# Dual-mode dopamine increases mediated by 5-HT_1B_ and 5-HT_2C_ receptors inhibition, inducing impulsive behavior in trained rats

**DOI:** 10.1007/s00221-019-05611-1

**Published:** 2019-07-27

**Authors:** Taizo Nakazato

**Affiliations:** Department of Physiology, Juntendo University School of Medicine, 2-1-1 Hongo, Bunkyo-ku, Tokyo, 113-0033 Japan

**Keywords:** Dopamine, Eating disorder, Impulsive behavior, Serotonin, Striatum, Voltammetry

## Abstract

Patients with eating disorders exhibit problems with appetitive impulse control. Interactions between dopamine and serotonin (5-HT) neuron in this setting are poorly characterized. Here we examined 5-HT receptor-mediated changes in extracellular dopamine during impulsive appetitive behavior in rats. Rats were trained to perform a cued lever-press (LP) task for a food reward such that they stopped experiencing associated dopamine increases. Trained rats were administered the mixed 5-HT_1B/2C_-receptor antagonist metergoline, the 5-HT_2A/2C_-receptor antagonist ketanserin, and *p*-chlorophenylalanine (PCPA). We measured dopamine changes in the ventral striatum using voltammetry and examined the number of premature LPs, reaction time (RT), and reward acquisition rate (RAR). Compared with controls, metergoline increased premature LPs and shortened RT significantly; ketanserin decreased premature LPs and lengthened RT significantly; and PCPA decreased premature LPs, lengthened RT, and decreased RAR significantly. Following metergoline administration, rats exhibited a fast phasic dopamine increase for 0.25–0.75 s after a correct LP, but only during LP for an incorrect LP. No dopamine increases were detected with ketanserin or PCPA, or in controls. After LP task completion, metergoline also caused dopamine to increase slowly and remain elevated; in contrast, ketanserin caused dopamine to increase slowly and decrease rapidly. No slow dopamine increase occurred with PCPA. Inhibition of 5-HT_1B_- and 5-HT_2C_-receptors apparently induced dual modes of extracellular dopamine increase: fast phasic, and slow long-lasting. These increases may be associated with the suppression of acquired prediction learning and retention of high motivation for reward, leading to impulsive excessive premature LPs.

## Introduction

Impulsive appetitive behavior is a key symptom in autism spectrum disorder, eating disorders, and obsessive–compulsive disorders (OCD). Miyazaki et al. (2012a, b) demonstrated that patience to wait for rewards requires serotonin (5-HT) neurons. Moreover, 5-HT reuptake inhibitors (SSRIs) are useful for treating pathologic impulsive aggression (Armenteros and Lewis 2002; Hollander et al. 2000), suggesting that 5-HT-mediated processes are involved in controlling impulse-related behavior. However, SSRIs also cause impulsivity and aggression in humans (Spigset 1999). Overall, the actions of 5-HT are complex and insufficiently understood.

Multiple studies implicate 5-HT in feeding. For example, 5-HT_1,2_-receptor (R) agonists cause hypophagia (Park et al. 1999), and the 5-HT-depleting drug *p*-chlorophenylalanine (PCPA) causes hyperphagia (Chance et al. 1982; Dourish et al. 1986; Holmes et al. 1990). Moreover, the 5-HT_1B_-R reportedly plays a highly selective role in the modulation of offensive aggression (Olivier 2004), and 5-HT_1B_-R knock-out mice exhibit impulsive behavior (Bouwknecht et al. 2001; Saudou et al. 1994). In animal models of OCD, 5-HT_2C_-R activation can induce compulsive behavior (Flaisher-Grinberg et al. 2008; Graf et al. 2003; Tsaltas et al. 2005); however, other reports demonstrate that 5-HT_2C_-R activation decreases cocaine-seeking behavior (Cunningham et al. 2011) and reduces impulsivity in studies of anxiety (Martin et al. 2002). Furthermore, a 5-HT_2A_-R antagonist suppresses impulsivity in the 5-choice task, while a 5-HT_2C_-R antagonist leads to increased impulsivity (Robinson et al. 2008). Thus, the functions of 5-HT receptors remain enigmatic and poorly characterized.

In Parkinson’s disease patients, hyperactive and compulsive behaviors (e.g., pathologic gambling and shopping, hypersexuality) can be devastating complications of treatment with dopamine (DA)-R agonists (Hassan et al. 2011; Pontone et al. 2006; Weintraub et al. 2010), indicating that these behaviors are involved in activation of DA system. There have also been reports that impulsive behavior is related to the accumbens (ACC) DA neuron (Diergaarde et al. 2008; Goodman et al. 2010; Huff et al. 2010; Parsons and Justice 1993). However, there are controversial reports concerning ACC 5-HT neurons. Some say that 5-HT is related to the suppression of DA release and impulsive behavior (Muramatsu et al. 1988; Nakazato 2013); others note that 5-HT is related to stimulation of DA release and impulsive behavior (Jacocks and Cox 1992; Nurse et al. 1988; Spigset 1999). Meanwhile, ACC has demonstrated involvement in feeding behavior via the lateral hypothalamus (Grignaschi et al 1995; Rorabaugh et al. 2014; Stratford 2005) and is also involved in the OCD circuit (Huey et al. 2008; McCracken and Grace 2007). It is well-known that patients with eating disorders show depressive mood, as well as impulsive and compulsive behavior, and SSRIs are effective for them. To elucidate the mechanism of eating disorder, it is important to examine interactions between DA and 5-HT neurons during impulsive appetitive behavior.

Regarding neuronal firing in DA neurons, Schultz (2001) proposes three types of temporal modes: fast phasic changes related to reward prediction error (Kagohashi et al. 2008; Nakazato 2005; Schultz 1998); intermediate slow changes related to fear stress and food reward; and tonic changes related to enabling cognition and movement in parkinsonism (which are poorly characterized). We recently reported that 5-HT neurons exhibit dual modes of change during adaptation to the anxiety environment: slow phasic changes and tonic increases (Nakazato 2013). Our present study investigated the neuronal mechanisms of eating disorders by examining the interaction between DA and 5-HT neurons during impulsive appetitive behavior in rats. Rats were trained to perform a cued lever-press (LP) task for a food reward (Brimberg et al. 2007), and were administered serotonergic drugs, including the mixed 5-HT_1B/2C_-R antagonist metergoline (Maurel et al. 1998), the selective 5-HT_2A/2C_-R antagonist ketanserin, and the serotonin depletory *p*-chlorophenylalanine (PCPA) (Buchanan et al. 1994; Lee and Clifton 1992). We used voltammetry to examine the changes in extracellular DA in chronically electrode-implanted ventral striatum (Kagohashi et al. 2008; Nakazato and Akiyama 1999, 2002), and analyzed behavioral changes including premature LP, reaction time (RT), and reward acquisition rate (RAR).

## Methods and materials

### Animal care

Rats were housed at constant room temperature and humidity in a facility with a 12-h light/dark cycle. They were housed individually in home cages (710 × 460 × 315 mm; Ferplast, Italy). Animals were administered about 20 g of food (MF, Oriental Yeast Co., Ltd, Japan) on no-training or experimental days, and about 15 g on training days, after training was completed. Water was available ad libitum.

Experiments were performed according to the Principles of Animal Experiments at Juntendo University and approved by the Committee of Animal Experiments at Juntendo University.

### Operation and preparation for voltammetric measurements

At the time of operation, the rats weighed 380–430 g. They were anesthetized with an i.p. injection of pentobarbital (50 mg/kg) and held stationary in a stereotaxic apparatus. To measure extracellular DA, a carbon fiber electrode (diameter 7 µm) was implanted unilaterally in the right ventral striatum with the following coordinates: 1.0 mm anterior to the bregma, 2.5 mm lateral to the sagittal suture, 5.5 mm ventral to the cortical surface (Paxinos and Watson 1986). The procedure has been described previously in detail (Nakazato and Akiyama 1999).

All of the electrodes were initially activated (polished) in vitro for 4–5 h as previously described (Akiyama et al. 1985; Nakazato et al. 1988). Next, sensitivity to DA was examined. Electrodes with good sensitivity were selected and implanted into the rat brains, with 10 nM DA corresponding to 76.38 ± 4.97 pA (*n* = 12) in vitro.

### Experimental protocols

#### A cued LP reward task

**Fig. 1:**
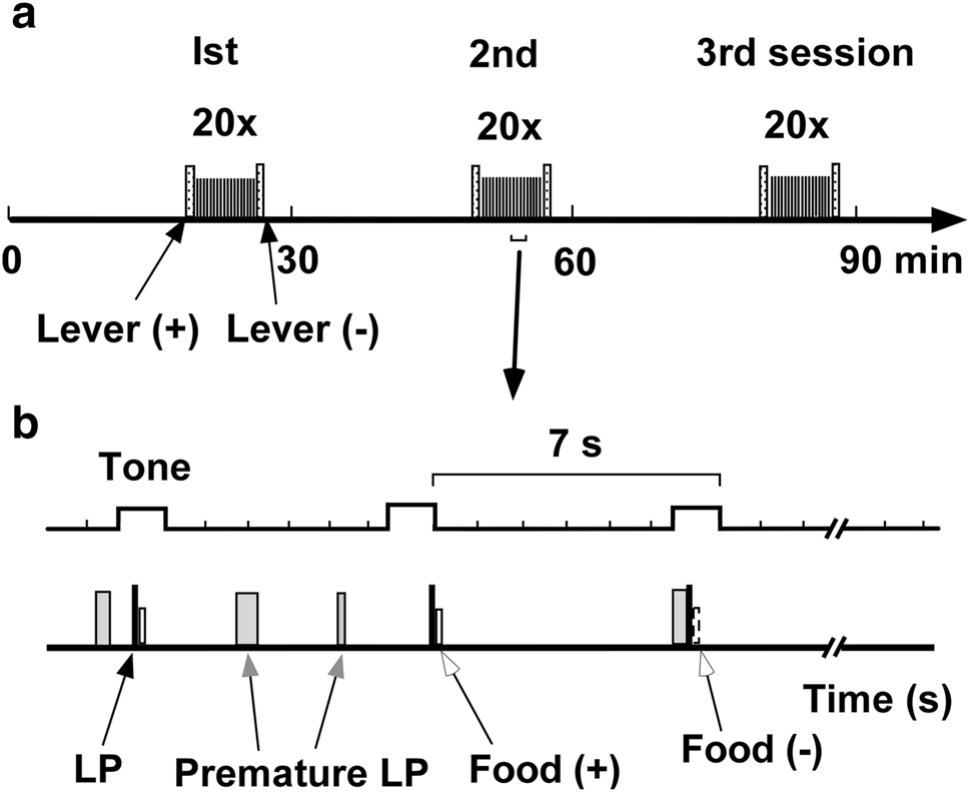
Experimental paradigm. **a** “Lever (+)” indicates lever appearance and “Lever (−)” indicates lever removal. The small bar between lever appearance and removal indicates sounding of the tone cue, which occurred 20 times per session. **b** Expansion of the intra-session paradigm. Closed bars indicate “correct” lever press (LP), open bars indicate food delivery, and grey bars indicate premature LPs. Bar thickness represents the duration of each continued LP. The first LP following a cue tone was considered correct and rewarded with food pellet delivery. LPs other than the correct LP were considered incorrect premature LPs, and were not rewarded with food delivery

Rats were trained to press a lever after hearing a tone to obtain a food reward (*n* = 6; 400–450 g). The details are described in a previous paper (Nakazato 2005). Briefly, the rats’ food intake was limited to 20 g/day, permitting post-operative weight gain of approximately 5 g/week. They were placed in a Skinner box (Muromachi Kikai Co. Ltd, Japan) equipped with a retractable lever. A cue tone (2.8 kHz; 1.5-s duration) was delivered every 7 s, with 20 tones per session (Fig. 1a). In an experimental day, eight sessions were performed at 30-min intervals. When the lever was pressed correctly, one 45-mg precision food pellet was provided approximately 200 ms later, and the same tone again sounded for 0.25 s. A correct LP was defined as the first LP occurring after the cue tone. Additional LPs before the next cue tone were considered errors (premature LP) and were not rewarded (Fig. 1b). Rats could obtain only one food reward during the 7 s between cues. LP training was performed 2–3 times weekly. Rats trained for more than 24–36 training days after operation were used for further examinations. Although the number of training days differed among rats, it was confirmed that DA increases did not occur with food rewards upon LP (Nakazato 2005), and these rats were considered trained.

#### Untrained condition

We also examined rats that were not trained for the LP task (*n* = 6; 400–450 g). These rats were reared individually in home cages, with food and water available ad libitum. Food and water were removed approximately 2 h before examination. A large food pellet (MF; 3.5–4 g, 12 mmφ) was placed in the home cage, and the rats’ behavior and extracellular DA changes were examined.

### Measurement of extracellular DA concentration

#### Conventional voltammetry

We measured DA concentration during the LP task using a conventional wired voltammetric system, which was described previously in detail (Nakazato and Akiyama 1999; Oyama et al. 2010). Briefly, the potential paradigm comprised a square-wave activation pulse (± 2000 mV) followed by a single-step measurement pulse. During measurements, 4 Hz activation and measurement pulses were applied. To measure DA, the potential was maintained at 100 mV, and then increased to 250 mV. The DA measurement sensitivity and differentiation of 5-HT and other compounds were described previously (Kagohashi et al. 2008; Nakazato and Akiyama 1999, 2002). Despite adsorption around the surface of the carbon fiber electrode (Atcherley et al. 2013), activation pulses enabled measurement of changes in DA signal currents even at high DA concentrations, because delivery of these pulses polished and renewed the electrode surface occupied with DA (Akiyama et al. 1985).

One week after the operation (implantation), the electrode was again activated due to low basal signal currents, likely caused by contamination of the electrode surface by bleeding at its insertion into the brain. The electrode was activated by delivering 5 Hz voltammetric pulses with the square activation pulse (± 2000 mV) for ≥ 5 days (for approximately 5 h/day) before the experiments (Nakazato 2005; Nakazato et al. 1988; Nakazato and Akiyama 1999).

#### Wireless voltammetry

Because the rats could move widely, we used wireless voltammetry to measure changes in DA concentration when untrained rats ate large pellets in the large home cage (710 × 460 × 315 mm). This procedure was described previously in detail (Kagohashi et al. 2008; Nakazato 2013). The electrode was also activated in the wireless system for ≥ 5 days before drug administration experiments (Kagohashi et al. 2008).

### Drugs

To investigate the influences of 5-HT antagonists, rats were i.p. injected with metergoline and ketanserin (*n* = 6 for each; doses 1 mg/kg) 30 min prior to starting the first session (Body et al. 2003; Lee and Clifton 1992; Maurel et al. 1998). These drug experiments were performed at least 1 week apart in the same rats. To investigate the effect of inhibiting presynaptic 5-HT release, 200 mg/kg PCPA (*n* = 6) was administered for 3 consecutive days (Holmes et al. 1990), and examinations of behavior and DA changes were initiated approximately 4 h after the last injection. This drug was administered 1–2 weeks after experiments with metergoline and ketanserin were completed.

### Measurements of behavioral changes

When multiple LPs were performed between a correct LP and the next cue tone, they were recorded as premature LPs. When a rat pressed the lever continuously, it was counted as one LP. RT was measured as the time from cue tone delivery to a correct LP, not counting any LPs within 0.25 s after the cue tone. We also calculated RAR (%), i.e., the rate of successful LPs.

### Histology

After completion of the experiments, the animals were killed under deep anesthesia. The head assembly (Nakazato and Akiyama 1992) was removed, and their brains were collected. These were soaked in phosphate-buffered buffered formalin and were embedded in paraffin. These were sliced in 5-µm thickness, and stained with cresyl violet just to confirm the electrode tip position.

### Data analysis

An experimental day included eight sessions of the LP task. However, data from the first two sessions were not used for analyses due to slight initial instability of the electrode for DA measurement.

To examine the fast phasic DA change, data were aligned at the rewarded LP. We averaged the data obtained for one rat over six sessions on an experimental day and then averaged the data for all examined rats. To examine a slow DA change, we measured the increase in DA concentration as follows: [average DA signal current for 30 s around the peak level for six sessions] or [average DA signal for 30 s at around 8 min after starting the task] − [average DA signal for 30 s just before starting the task]. These values were averaged for all examined rats. In untrained rats, the DA increase was measured as follows: [average DA current for 30 s just before the start of eating] or [average DA current for 30 s at 2 or 4 min after the start of eating] − [average DA current for 30 s just before food presentation]. These values were averaged for six rats.

Control data were obtained during the previous experimental day immediately before tests with drugs. Data were analyzed using a paired *t* test or Welch’s *t* test, and Scheffe’s test after one-way or two-way ANOVA. A *p* value < 0.05 was considered statistically significant.

## Results

**Fig. 2:**
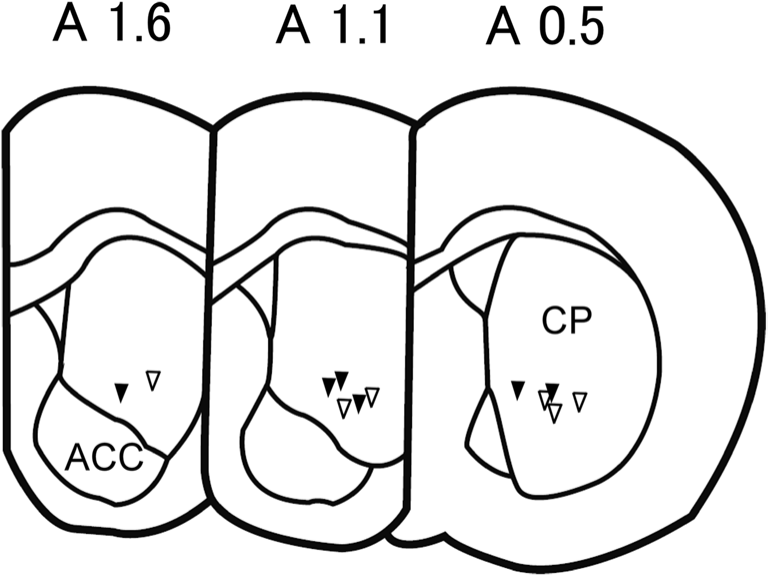
The positions of the tips of recording electrodes. Recordings were obtained from examined rats using conventional (closed triangles) or wireless (open triangles) voltammetry. Figures were modified from the rat brain atlas (Paxinos and Watson 1986)

The tips of the recording electrodes were found in the ventral striatum (Fig. 2).

### Behavioral changes after drug administration

**Fig. 3:**
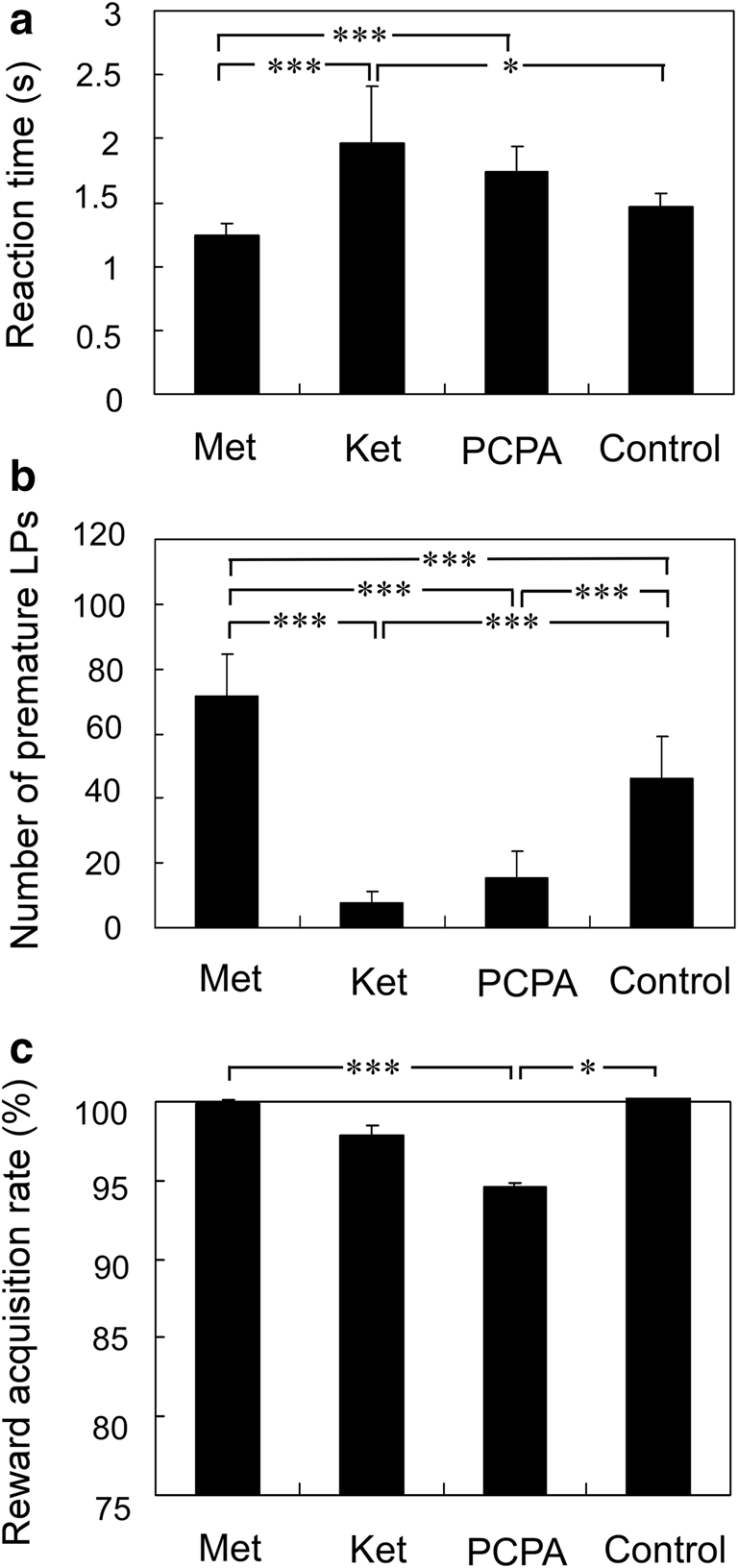
Comparisons between metergoline, ketanserin, PCPA, and controls regarding reaction time (RT), number of premature lever presses (LPs), and reward acquisition rate (RAR). **a** RT differed significantly between metergoline and ketanserin, between metergoline and PCPA, and between ketanserin and control. **b** The number of premature LPs differed significantly between metergoline and ketanserin, between metergoline and PCPA, between metergoline and control, between ketanserin and control, and between PCPA and control. **c** RAR differed significantly between metergoline and PCPA, and between PCPA and control. Data are presented as mean ± SD. *p < 0.05, ***p < 0.01, by Scheffe’s test

To clarify the different effects of each drug on behavior, we performed multiple comparisons between metergoline, ketanserin, PCPA, and controls. Data for each control were obtained during the day before drug administration. RT differed significantly between metergoline and ketanserin (Fig. 3a; *p* < 0.01, Scheffe’s test), between metergoline and PCPA (*p* < 0.05), and between ketanserin and control (*n* = 18; *p* < 0.01). The number of premature LP differed significantly between metergoline and ketanserin, between metergoline and PCPA, between metergoline and control, between ketanserin and control, and between PCPA and control (Fig. 3b; *p* < 0.01 for each pair by Scheffe’s test). The RAR was 99.83 ± 0.37% with metergoline, 97.89 ± 1.39% with ketanserin, 94.5 ± 3.23% with PCPA, and 99.7 ± 0.39% in controls (Fig. 3c). RAR differed significantly between metergoline and PCPA (*p* < 0.01, Scheffe’s test) and between PCPA and control (*p* < 0.01), suggesting that PCPA caused a significant decrease in RAR.

### Extracellular DA changes after drug administration

#### Fast DA change upon a rewarded LP

**Fig. 4:**
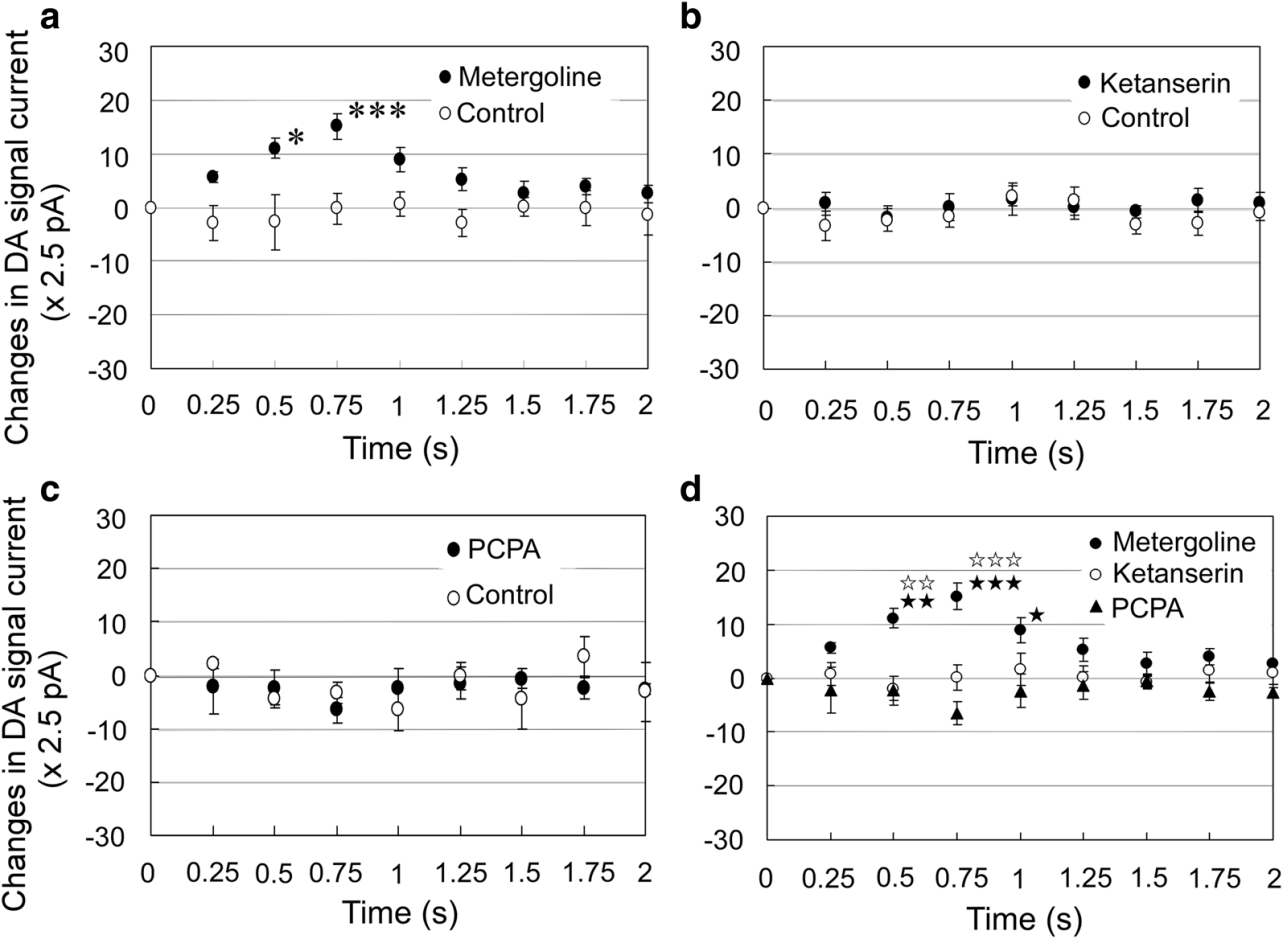
Fast phasic dopamine (DA) changes upon rewarded lever presses (LPs)  after drug administration. Data were aligned at the time of LP (time 0) and compared with control data obtained in rats the day before drug administration. **a** Metergoline administration (filled circle; *n* = 6) caused significant fast DA increases (Scheffe’s test; *p *< 0.01), and the metergoline group differed significantly from the control group (open circle; *n* = 6; *p* < 0.03, two-way ANOVA). Compared to time 0, the metergoline group showed significant increases at 0.5 s and 0.75 s (*p < 0.05, ***p < 0.01, Scheffe’s test). **b** The ketanserin group (filled circle; *n* = 6) did not show significant changes compared with the control group (open circle; *p* = 0.60, two-way ANOVA). **c** PCPA did not cause significant fast phasic DA increases (*p* = 0.94; *n* = 6). **d** Simultaneous comparisons between fast DA changes after administration of metergoline (filled circle), ketanserin (open circle), and PCPA (filled triangle) are shown. Changes after metergoline administration differed significantly from changes after ketanserin or PCPA administration (both *p* < 0.01, two-way ANOVA). No significant difference was found between ketanserin and PCPA (*p* = 0.92). Significant differences were found at 0.5 s between metergoline and ketanserin (^☆☆^*p *< 0.03, Welch’s *t* test) and between metergoline and PCPA (^★★^*p *< 0.03); at 0.75 s between metergoline and ketanserin (^☆☆☆^*p *< 0.01) and between metergoline and PCPA (^★★★^*p *< 0.01); and at 1.0 s between metergoline and PCPA (^★^*p *< 0.05). Data are presented as mean ± SE

Data were aligned with LP as the time 0. Metergoline administration caused a fast phasic DA increase in the ventral striatum after LP (Fig. 4a), with significant increases between time 0 and 0.5 s (*p* < 0.5, Scheffe’s test) and between time 0 and 0.75 s (*p *< 0.01). The metergoline group significantly differed from control group (*p* < 0.03, two-way ANOVA). On the other hand, the ketanserin group did not significantly differ from controls (*p* = 0.60, two-way ANOVA; Fig. 4b). PCPA did not cause significant fast DA changes (*p* = 0.94; Fig. 4c). To clarify the differences between changes, we performed multiple comparisons between metergoline, ketanserin, and PCPA (Fig. 4d). The changes after metergoline administration were significantly different from those after ketanserin and PCPA administration (both *p* < 0.01, two-way ANOVA). No significant difference was observed between ketanserin and PCPA (*p* = 0.92). Significant differences were seen at 0.5 s between metergoline and ketanserin (*p *< 0.03, Welch’s *t* test) and between metergoline and PCPA (*p *< 0.03); at 0.75 s between metergoline and ketanserin (*p *< 0.01) and between metergoline and PCPA (*p *< 0.01); and at 1.0 s between metergoline and PCPA (*p *< 0.05).

#### Fast DA change upon an unrewarded premature LP

**Fig. 5:**
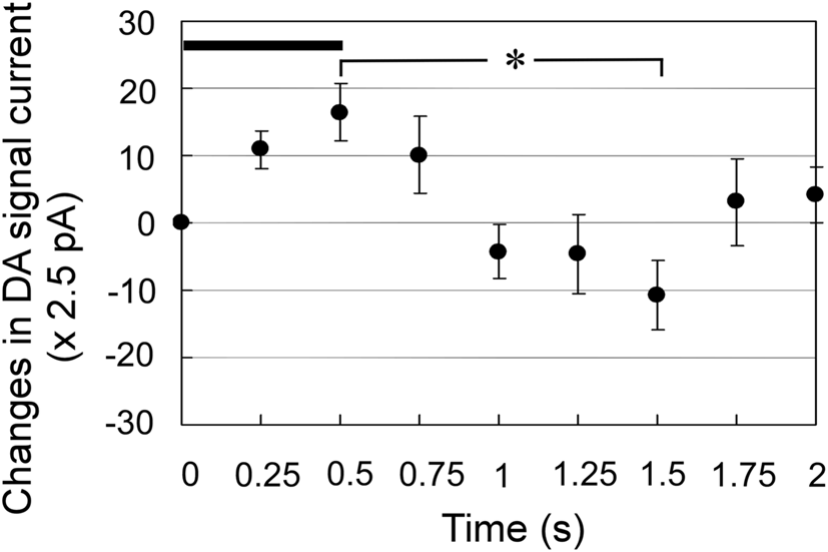
Fast dopamine (DA) changes upon unrewarded premature lever presses (LPs) after metergoline administration. Mean changes (± SE) of DA concentrations in six rats are shown. Examined data (*n* = 14) include instances where the incorrect lever press lasted ≥ 0.5 s, and there was a latent time of > 2 s before the next cue-tone delivery. We found a significant difference between 0.5 and 1.5 s (*p < 0.05, Scheffe’s test). DA increases were reduced soon after lever release. DA appeared to increase again starting at 1.75 s, although this change was not significant. Horizontal bar indicates the duration of incorrect lever press

After metergoline administration, a DA change was detected upon an incorrect unrewarded premature LP. Figure 5 shows analysis of DA changes in instances where the duration of incorrect lever-pressing was 0.5 s and the time from lever release to delivery of the next cue tone delivery exceeded 2 s. Notably, such data were scarce, with 14 such occurrences among 6 rats (1–4 LPs per rat). However, we identified a significant difference between 0.5 s and 1.5 s (*p* < 0.05, Scheffe’s test), indicating that DA increases were significantly reduced soon after lever release. Moreover, DA seemed to increase again from 1.75 s, although this change was not significant.

Very few incorrect LPs were performed after the administration of ketanserin or PCPA, such that data analysis could not be performed.

#### Slow DA change in trained rats

**Fig. 6:**
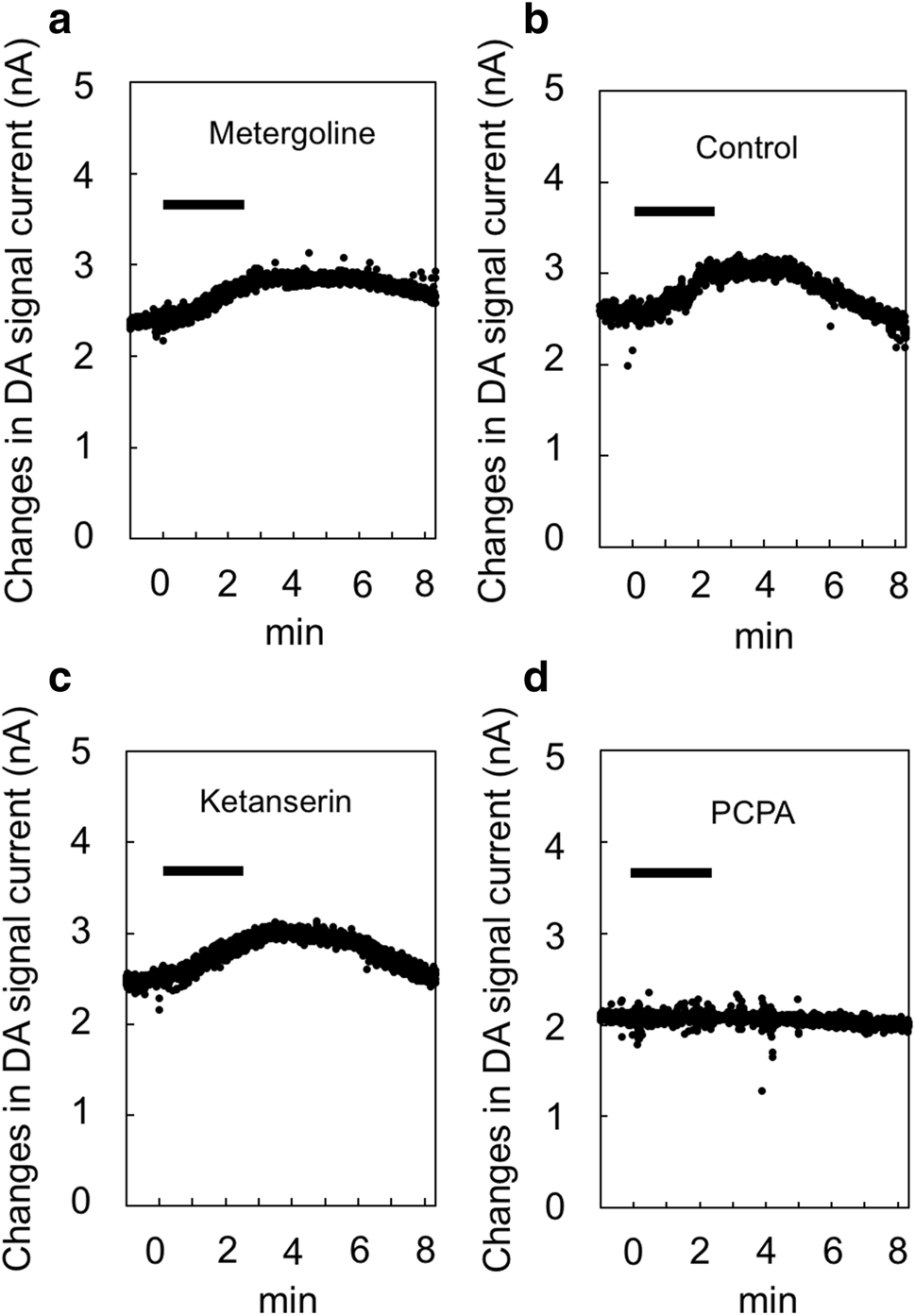
Slow dopamine (DA) changes during the lever press (LP) task after drug administration. A representative case is shown for each drug and for the control. **a** When metergoline was administered, DA increased gradually after the start of the task, peaked near the end of the session or later, and **b** remained high for > 8 min compared with the control. **c** Ketanserin caused a slow DA increase, which did not last > 8 min. **d** PCPA did not cause a slow DA increase. The horizontal bar indicates the LP task duration within a session

We also examined slow DA changes during the LP task after drug administration. In addition to phasic DA change after metergoline administration, a slow DA increase was also observed simultaneously, which peaked near the end of the session (Fig. 6a). The slow DA increase with metergoline remained higher and lasted more than 6 min after LP task completion, longer than with the drug-free control (Fig. 6b). We also noted a slow DA increase with ketanserin; however, it did not last more than 6 min (Fig. 6c). Eight minutes after the start of the task, the DA level reached basal levels in ketanserin injection and control. To compare clearly the differences between DA and metergoline time courses, 8 min is the maximum on the abscissa. No obvious increase was observed after PCPA injection (Fig. 6d).

**Fig. 7:**
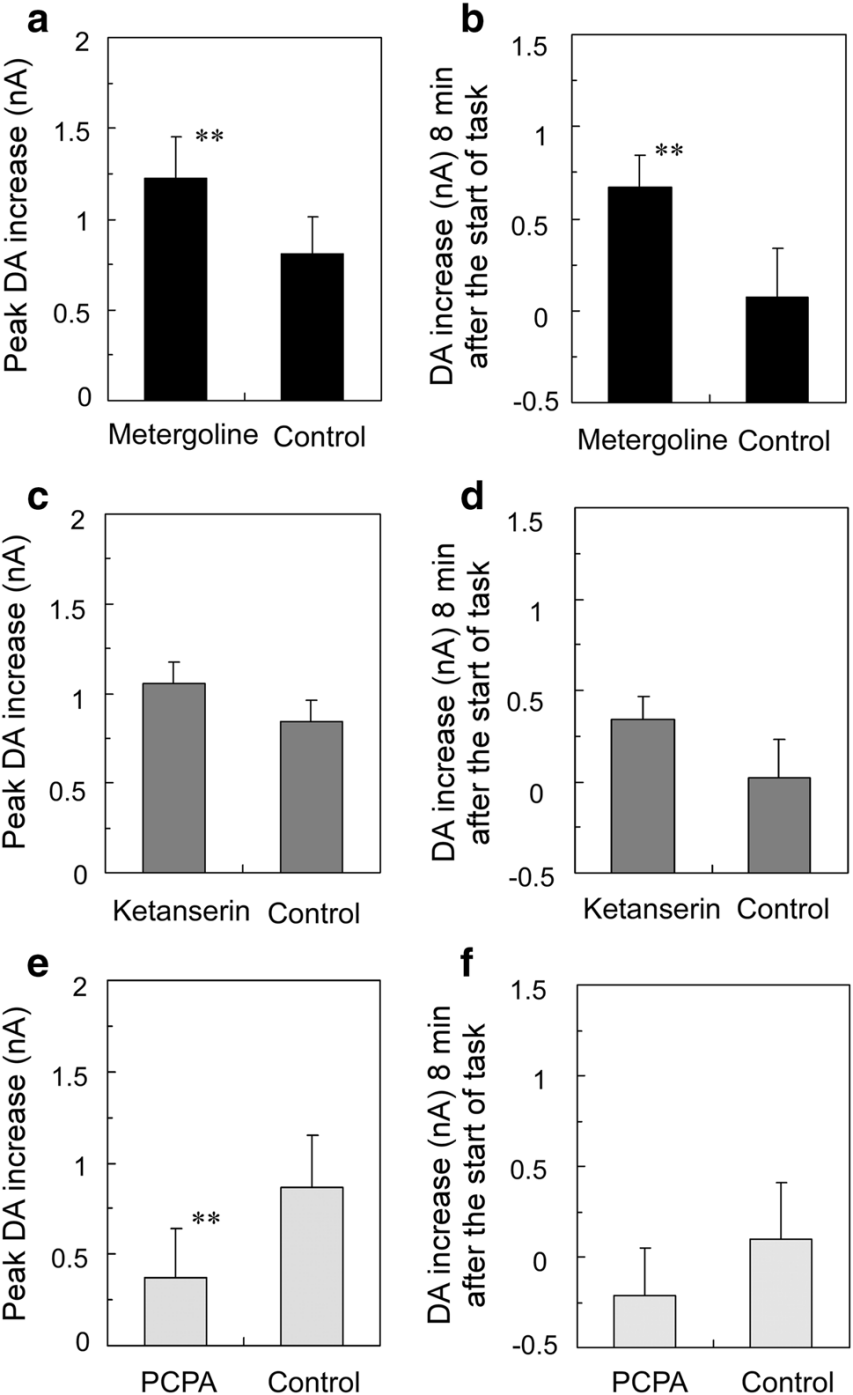
Slow dopamine (DA) increases (mean ± SD) at the peak of DA increase and at 8 min after the start of the lever press task, after administration of metergoline (closed box), ketanserin (dark gray box), and PCPA (light grey box). The increases were compared with control data obtained on the previous experimental day without drugs. **a**, **b** Compared to controls, metergoline administration (*n* = 6) led to significantly increased DA levels at peak and 8 min after the start of the task. **c**, **d** After ketanserin administration (*n* = 6), the peak and 8-min DA levels did not differ significantly from controls (*p* = 0.08 and 0.07, respectively). **e**, **f** After PCPA administration (*n* = 6), the peak DA increase was suppressed significantly compared to controls, while the 8-min DA level did not differ significantly from controls (*p* = 0.18). **p < 0.03, paired *t* test

To examine the characteristics of the observed DA changes, we calculated the average peak DA levels and the average DA levels at 8 min after the start of the LP task (Fig. 7). Control data (*n* = 6) were obtained for each rat in the previous drug-free examinations. After metergoline administration, the peak DA level and the average DA level at 8 min after LP task initiation were significantly higher than controls (*p* < 0.03, paired *t* test; Fig. 7a, b). The peak DA level and the average DA level 8 min after LP task initiation were not significantly different from controls after ketanserin administration (*p* = 0.08 and 0.07, respectively; Fig. 7c, d). After PCPA administration, the peak DA increase was significantly suppressed compared with control (*p* < 0.03, paired *t* test; Fig. 7e), while the average DA level 8 min after LP task initiation did not significantly differ from control (*p* = 0.18; Fig. 7f).

#### Slow DA change in untrained rats

**Fig. 8:**
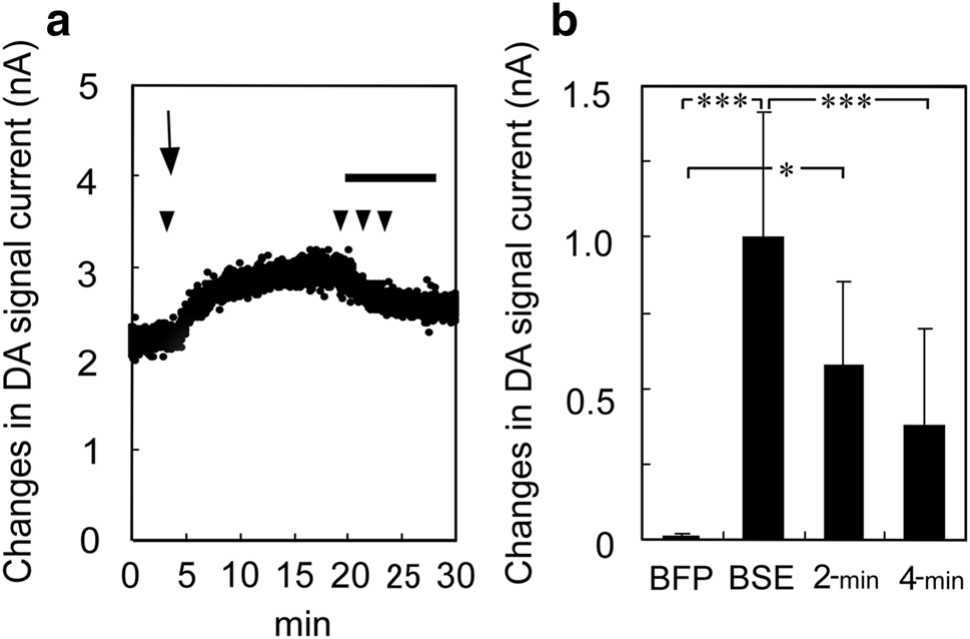
Slow dopamine (DA) changes in untrained rats (*n* = 6) upon presentation of a large food pellet. **a** A representative case is shown. Upon placement of a food pellet in the cage, DA increased slowly until the start of eating and then decreased soon after the end of eating. **b** These DA changes were examined in six rats. DA levels differed significantly just before food presentation versus just before the start of eating (*p* < 0.01, Scheffe’s test), just before the start of eating versus at 4 min after the start of eating (*p* < 0.01), and just before food presentation versus at 2 min after the start of eating (*p* < 0.05). DA current just before food presentation was defined as 0 current. Arrow indicates the start of eating. Horizontal bar indicates the duration time of eating. The data indicated by the closed arrow-heads correspond to the results shown in **b**. *BFP* just before food presentation, *BSE* just before the start of eating, *2-min* 2 min after the start of eating; *4-min* 4 min after the start of eating. *p < 0.05, ***p < 0.01

For investigations in untrained rats, a large pellet was placed in the cage, and DA changes were examined. The interval from food presentation to the start of eating, and the time taken for food consumption, differed among the rats (*n* = 6); Fig. 8a shows a representative case. DA began to increase slowly after food presentation, peaked near the start of eating, and decreased soon after the start of eating. The mean DA levels for the six rats differed significantly just before food presentation versus just before the start of eating (*p* < 0.01, Scheffe’s test), just before the start of eating versus at 4 min after the start of eating (*p* < 0.01), and just before food presentation versus at 2 min after the start of eating (*p* < 0.05; Fig. 8b). DA current just before food presentation was defined as 0 current.

## Discussion

### Behavioral changes during LP task in trained rats

#### LP behavior and 5-HT-Rs

Our results indicated that premature LPs were greatly decreased by ketanserin and greatly increased by metergoline. Ketanserin is a selective 5-HT_2A/2C_ antagonist that blocks 5-HT_2A_-R more potently than 5-HT_2C_-R (Body et al. 2003; Sander-Bush and Mayer 2006). Metergoline is a mixed 5-HT_1B/2C_-R antagonist (Maurel et al. 1998). Release of 5-HT is reportedly inhibited via the 5-HT_1B_ auto-R (Engel et al. 1986; Sarhan et al. 2000). Thus, our results may indicate that 5-HT_2A_-R inhibition suppressed premature LPs and that inhibition of 5-HT_1B_-R and 5-HT_2C_-R was implicated in increasing premature LPs. We found that PCPA administration decreased premature LPs and RAR. There have been reports that PCPA prevents presynaptic auto-R mediated inhibition in 5-HT neurons (Dourish et al. 1985), that stimulation of the 5-HT_2C_-R reduces motivation (Valencia-Torres et al. 2017), and that a 5-HT_2C_-R antagonist leads to increased impulsivity (Robinson et al. 2008). These reports are not contradictory to the above-mentioned idea.

#### Excessive premature LPs and impulsive behavior

Serotonin systems are implicated in incentive motivation for cocaine, and 5-HT_1B_-R stimulation produces a general decrease in motivation (Acosta et al. 2005; Pentkowski et al. 2009). In mice, 5-HT_1B_-R knock-out leads to impaired impulse control (Bouwknecht et al. 2001; Saudou et al. 1994). In humans, 5-HT_1_-R and 5-HT_2_-R are reportedly implicated in OCD pathophysiology (Tsaltas et al. 2005), while 5-HT_1_-R and 5HT_2A_-R agonists can cause anorexia and hypophagia (Kennet et al. 1987; Park et al. 1999). Lee and Clifton (1992) demonstrated that fluoxetine-induced anorexia was antagonized by metergoline, but not by ketanserin. However, no studies indicate that metergoline causes hyperphagia. Our present results indicated that metergoline injection shortened RT in trained rats. Overall, the available data indicate that metergoline likely elicits impulsive appetitive behavior (Cheetham and Heal 1993) and that 5-HT_1B_-R and 5-HT_2C_-R antagonistic actions are implicated in this behavior.

PCPA-mediated 5-HT depletion can reportedly cause hyperphagia (Chance et al. 1982; Dourish et al. 1986; Holmes et al. 1990). However, we found that PCPA-treated rats showed significantly decreased premature LPs and significantly suppressed RAR compared to controls. In another study in rats, Paxinos et al. (1997) reported suppressed food intake on the day after the last PCPA injection, after which food intake subsequently recovered and increased. In birds, Buchanan et al. (1994) reported suppressed pecking on the last day of 3 consecutive days of PCPA administration. Most PCPA studies include examination over several days after 3 days of PCPA administration, and data suggest that PCPA causes appetite suppression on the last day of PCPA administration or on the next day (Paxinos et al. 1997). Moreover, Joel et al. (2004) reported that fluoxetine attenuated excessive LPs in a rat model of OCD. Thus, it is also possible that PCPA suppressed impulsive appetitive behavior and high motivation for reward.

### Dual modes of DA increase during the LP task in trained rats

#### DA increases and 5-HT-Rs

The 5-HT_1B_-R exists in the ventral striatum and ACC (Bonaventure et al. 1998). The 5-HT_1B_-R is a presynaptic auto-R (Varnäs et al. 2005). The 5-HT_2C_-R is reportedly located in the ventral tegmental area (VTA) (Bubar and Cunningham 2007), and its selective agonist SB 242084 exerts inhibitory influence postsynaptically on VTA DA neurons and tonically inhibits DA releases in the ACC (De Deurwaerdère et al. 2004). Our present results demonstrate that metergoline causes a fast phasic increase of DA in the ventral striatum of trained rats upon a rewarded LP, while no such fast DA increase was detected with ketanserin. Because ketanserin is more selective for 5-HT_2A_-R than for 5-HT_2C_-R, it may be concluded that a fast DA change primarily involved 5-HT_1B_-R rather than 5-HT_2C_-R. Furthermore, both metergoline and ketanserin caused slow DA increases, but only metergoline caused a long-lasting DA increase, possibly suggesting that 5-HT_2C_-R was the main mediator of long-lasting DA increase. 5-HT_2A_-R is observed in the frontal cortex (Pompeiano et al. 1994). Activation of this receptor induced an enhancement of excitatory postsynaptic currents (EPSP) (Huang et al. 2009). Cortical 5-HT_2A_-R activation augments glutamatergic transmission in the ACC (Mocci et al. 2014). Therefore, it may be suggested that inhibition of such a 5-HT_2A_-R-related system by ketanserin did not cause a fast DA increase, resulting in a decrease in premature LPs. Also, 5-HT_2C_-R antagonism by ketanserin is reportedly weak compared with 5-HT_2A_-R; however, its weak action is undeniably related in some way with the appearance of slow DA increase because it appears to be increased more than control, although not significantly so.

#### Fast phasic DA increases and reward learning

Serotonergic neurons exhibit diverse projections to the hypothalamus, paraventricular thalamic nucleus (PVT), and ACC (Moore et al. 1978). PVT cells project to ACC nuclei, which are related to feedings (Lee et al. 2015; Stratford 2005). Cone et al. (2014) demonstrated an increase in phasic ACC DA changes during pellet retrieval in food-restricted rats, indicating that ACC DA neurons are likely related to motivated feeding behavior. Our present experiments were performed using trained rats that no longer exhibited a fast phasic DA increase upon food reward (Nakazato 2005), consistent with findings that DA neurons are involved in prediction error (Kawagoe et al. 1998; Schultz 1998). We found that metergoline injection caused a fast DA increase after LP in the trained rats, likely indicating the suppression of acquired prediction LP learning, with consequent induction of DA increase for an unexpected reward.

In contrast, ketanserin administration led to a substantial decrease in premature LPs and no significant change in RAR. These findings suggested that ketanserin did not influence appetite. Together with the lack of a fast DA increase after ketanserin injection, it seems likely that the 5-HT_2A_-R antagonist has suppressive effects on impulsive behavior without influencing acquired LP learning. Consistent with this interpretation of our findings, Robinson et al. (2008) previously reported that the 5-HT_2A_-R antagonist M100907 suppresses impulsivity in the 5-choice serial reaction time task.

In instances of unrewarded incorrect LPs, DA increase occurred only during a lever-pressing (Fig. 5) after metergoline injection. In these circumstances, DA decreased soon after the lever was released with no reward, in contrast to the post-LP DA increase in the case of rewarded correct LPs. DA increase has been reported in cocaine-desire among addicted rats (Kiyatkin and Stein^1994^). Phasic DA increase also occurs in the rat ventral striatum upon receipt of an unexpected reward (Nakazato 2005). These data suggest that the DA increases during LP are related to reward desire or expectation, while the DA increase after LP is related to prediction error.

#### Slow, long-lasting DA increases and motivation

Trained control rats did not exhibit a fast DA increase upon reward (Nakazato 2005); however, they did show a slow DA increase (Fig. 6b). Metergoline-injected trained rats exhibited both a fast phasic DA increase and a slow long-lasting DA increase, and the slow increase was higher than in control. In contrast, ketanserin-injected trained rats showed only slow short-term DA increases. When an experimenter put a large food pellet on the usual place in the home-cage of untrained rats, rats soon noticed the usual food; DA slowly increased until the start of eating, and then decreased shortly after that. This DA increase may indicate eagerness for food (i.e., intermediate or tonic DA change indicated by Schultz (2001)). Thus, the metergoline-induced long-lasting DA increases may suggest that the high motivation for reward was maintained. Notably, from the finding that DA increases were observed upon incorrect LPs, it also may be considered that the long-lasting DA increases could be due to extracellular DA diffusion related to excessive LPs. However, because such diffused DA quickly disappears from extracellular space (Nakazato and Akiyama 1988), the increases were more likely due to motivation-related DA. Covey et al. (2016) reported that the DA increase after the LP was on the order of 10 nM in mice. In our voltammetric system, in vitro, 10 nM DA corresponded to 76.38 ± 4.97 pA (*n* = 12). Considering these, the slow DA increase was higher than their results, although our data were obtained from the metergoline administration experiment. Meanwhile, the fast DA increase seemed similar in DA concentration. The slow DA changes in our experiment could not disprove contamination of the DA metabolite 3,4-dihydroxyphenylacetic acid (DOPAC) when the DOPAC concentration increased 100-fold higher than the DA concentration (Nakazato and Akiyama 1999). However, our idea concerning the slow changes is supported.

It was recently reported that appetitive consummatory behavior is caused by activation of ventral tegmental DA neurons via the lateral hypothalamus (Jennings et al. 2015; Nieh et al. 2015). This neuronal circuit may involve a slow DA increase.

### Clinical implications

Eating disorders, such as bulimia nervosa and binge-eating disorder accompanied with impulsive behavior, have become common societal problems. Moreover, pathological impulsive behaviors as serious complications of treatment with DA agonists are reported in patients with Parkinson disease, as well as in those with restless leg syndrome and pituitary adenoma (Moore et al. 2014; Voon et al. 2011). Doya (2002) reported that decreased 5-HT is implicated in impulsive behavior, in that a 5-HT increase suppresses DA neuron activity, resulting in the suppression of impulsive behavior. Our presently described experimental results after administration of 5-HT_1B_-R and 5-HT_2C_-R antagonists may support Doya’s proposal. Moreover, the prevention of presynaptic inhibition of 5-HT neurons using PCPA (Dourish et al. 1985) did not lead to increased DA in response to a food reward, likely suggesting that stimulation of 5-HT_1B_ auto-R suppressed impulsivity. From the available data, it may be concluded that impulsivity strength was determined based on dual modes of DA increase mediated via inhibition of 5-HT receptors. Thus, the development of agonists specific to both of 5-HT_1B_-R and 5-HT_2C_-R may provide a therapy for impulsive control disorder involving eating disorder. Moreover, a 5-HT_2A_-R-specific antagonist could also be useful for suppressing this behavior (Robinson et al. 2008).

## Conclusion

In trained rats, inhibition of the 5-HT_1B_ and 5-HT_2C_ receptors was found to cause excessive unrewarded LPs and was also found to induce dual modes of extracellular dopamine increase using voltammetry: fast phasic (< 2 s), and slow long-lasting (> 10 min). These DA increases were thought to be associated with suppression of acquired predictive learning and retention of high motivation for reward, respectively, resulting in excessive increases of unrewarded LPs. These experimental results may play a role in clarifying the mechanism underlying impulsive eating disorders and may be useful in developing therapeutic drugs.
